# Supplementary material for: Absorption-induced transmission in plasma microphotonics
Source: Nat Commun. 2023 Jul 27;14:4535. doi: 10.1038/s41467-023-40205-0 (PMC10374664; doi:10.1038/s41467-023-40205-0)
Supplement: Supplementary file 1 — Supplementary Information [file 41467_2023_40205_MOESM1_ESM.pdf]

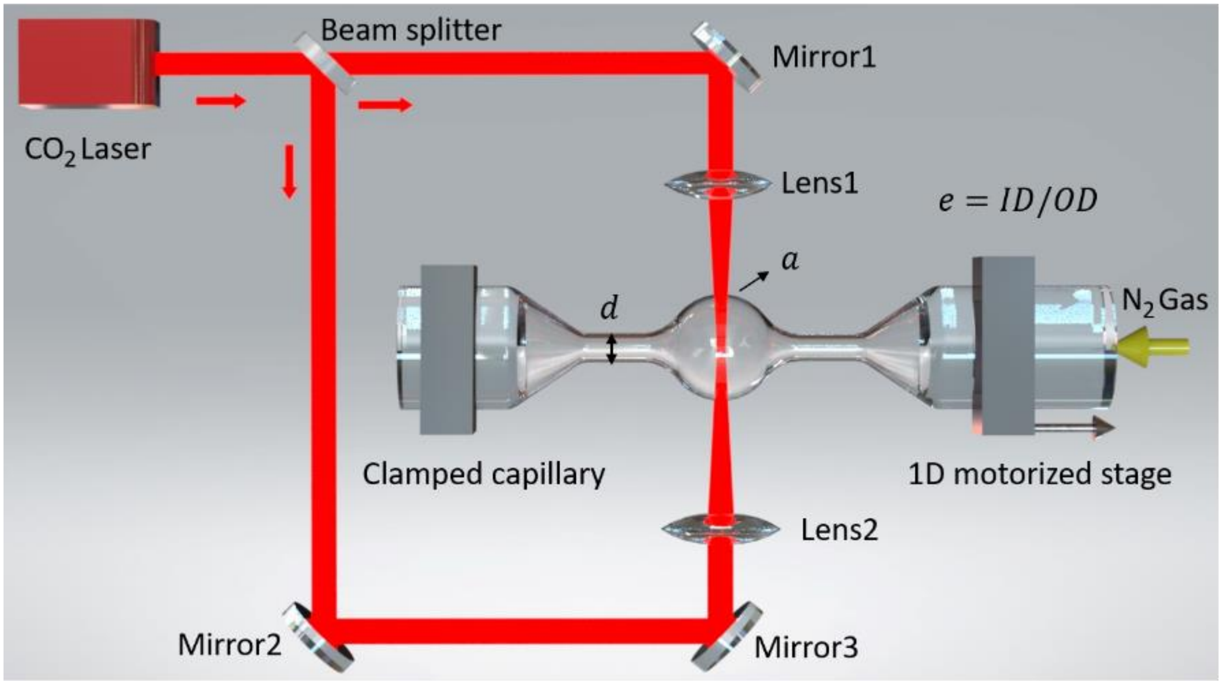

Supplementary Fig. 1 | Microbubble cavity fabrication. A clamped glass microcapillary was tapered and blown to form a microbubble using CO_2 laser light that was initially split to make two counter-propagating beams and focussed onto a section using a combination of mirrors and lenses. Nitrogen gas was used to pressurise the tapered capillary while heating the section using the CO_2 laser.

Supplementary Fig. 2 | IR micrographs of bubble modes next to their corresponding calculated modes where colours stand for light intensity.

Mode order	WGM IR Micrograph	Corresponding calculated mode
1st mode	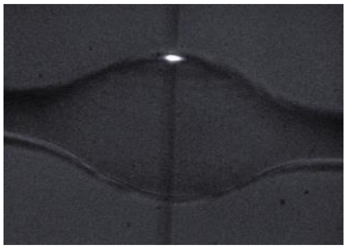	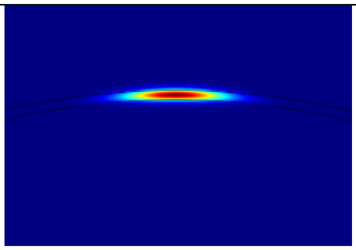
2nd mode	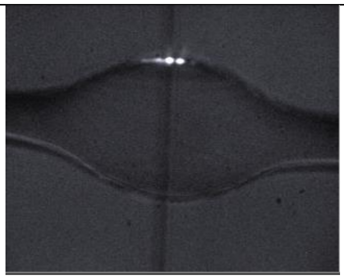	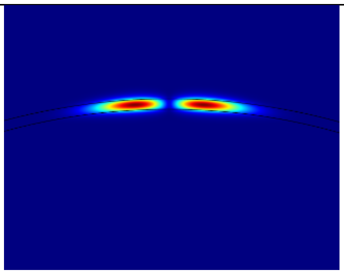
3rd mode	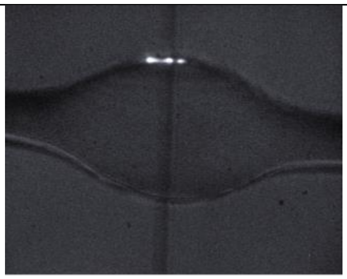	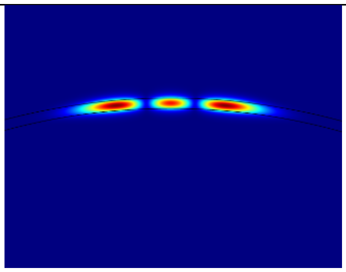
4th mode	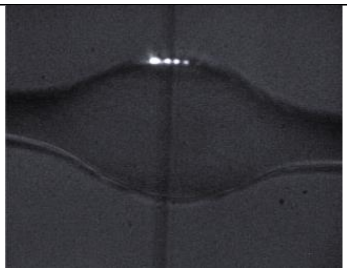	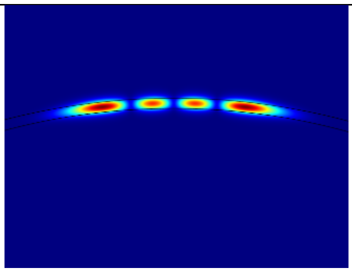
5th mode	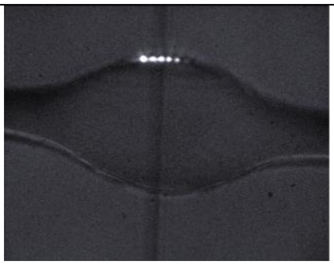	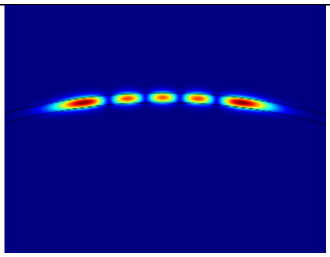

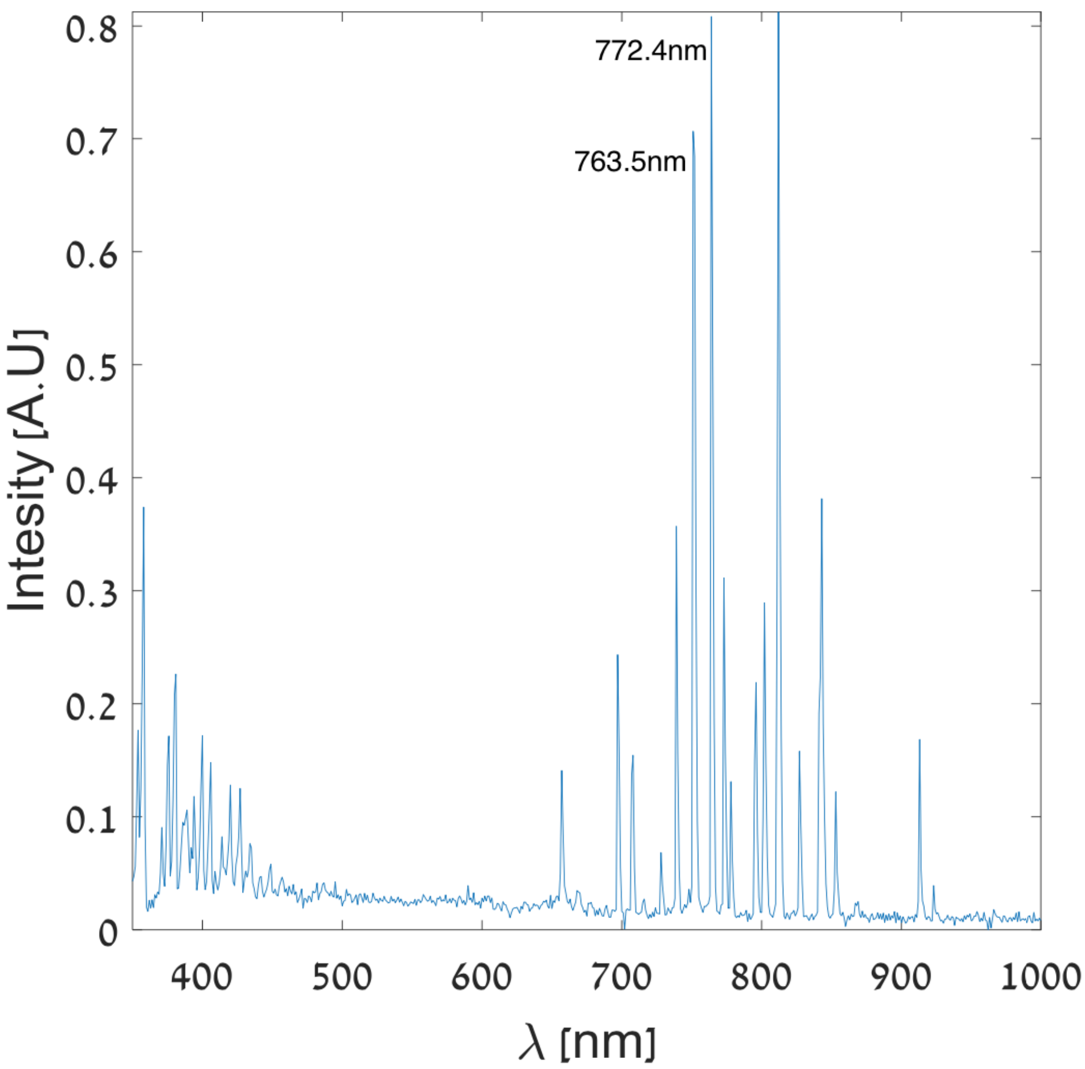

Supplementary Fig. 3 | Plasma emission spectrum as measured via free-space.
